# Associations between weight misperception, contextual factors, and weight loss behaviours in young adult men with overweight/obesity

**DOI:** 10.1002/osp4.382

**Published:** 2019-12-02

**Authors:** Andrew C. Pool, Donna L. Coffman, David B. Sarwer, Jessica G. LaRose, Chantelle N. Hart

**Affiliations:** ^1^ Center for Parent and Teen Communication Craig‐Dalsimer Division of Adolescent Medicine, Children's Hospital of Philadelphia Philadelphia Pennsylvania; ^2^ Department of Epidemiology and Biostatistics Temple University Philadelphia Pennsylvania; ^3^ Center for Obesity Research and Education Temple University Philadelphia Pennsylvania; ^4^ Department of Social and Behavioral Sciences Temple University Philadelphia Pennsylvania; ^5^ Department of Health Behavior and Policy Virginia Commonwealth University School of Medicine Richmond Virginia

**Keywords:** race differences, weight loss, weight perception, young adults

## Abstract

**Objective:**

Young men are less likely to engage in weight loss behaviours than their female counterparts. This may be because of an increased likelihood for young men, particularly young black men, with overweight/obesity to misperceive their weight status. This study examined racial differences in weight status perception accuracy and associations between this perception and weight loss behaviours among young men. Associations between weight loss behaviours and contextual factors were also explored.

**Methods:**

Data from 1417 young adult (YA) men with overweight/obesity from the 2007 to 2014 National Health and Nutrition Examination Survey (NHANES) were analysed. Associations between weight status perception accuracy, contextual factors, and weight loss attempts and behaviours were examined with logistic regression.

**Results:**

YA men with overweight/obesity were more likely to report weight loss attempts and behaviours if they perceived themselves as being overweight (OR = 3.10; 95% CI, 2.18‐4.41; *P* < .01; OR = 3.20, 95% CI, 2.16‐4.72, *P* < .01, respectively). Greater education and income were associated with a greater likelihood of reporting weight loss attempts and healthy weight loss behaviours. Greater reported depressive symptoms were associated with reported weight loss attempts but not healthy weight loss behaviours. There were no differences by race for reported weight loss attempts or behaviours.

**Conclusion:**

Among YA men with overweight/obesity, perceiving oneself as overweight was associated with reporting weight loss attempts and healthy weight loss behaviours. Future research should consider how weight status perception accuracy affects weight loss attempts among YA men and what additional factors may account for racial differences.

## 1 INTRODUCTION

Among young adults (YAs; 20 to 39 years of age), the prevalence of overweight/obesity, defined as a body mass index (BMI) of 25.0 to 29.9 kg/m^2^ and greater than or equal to 30.0 kg/m^2^, respectively, has increased significantly over the past 30 years, particularly among YA men.^1^, ^2^ National estimates indicate 62% of YA men have overweight/obesity.^2^ Further, individuals experience the greatest rate of weight gain during young adulthood, with white and black men experiencing average 5‐year gains of 9 and 13 pounds, respectively.^3^ This weight gain is associated with an increased risk of numerous obesity‐related comorbidities and early mortality, especially for black men.^1^, ^4^, ^5^

Several factors are likely responsible for this excess weight gain among YA men. Fruit and vegetable consumption among YA men has declined significantly since the 1980s,^6^ and YA men are not highly engaged in preparing their own food, which may lower the overall quality of their diets.^7^ Further, although young men consistently report higher levels of physical activity than YA women, they do not meet national recommendations.^6^ Importantly, YA men are less likely to engage in weight loss than are YA women.^8^ Unfortunately, little research has explored which factors could best explain why this may be the case. Given increased risk of weight gain during this time period as well as significant obesity‐related comorbidities for YA men in general,^9^ and black men specifically,^5^ it is important to understand why they are less likely to attempt weight loss.

One factor that may be important is the accuracy with which a YA male perceives his weight status. Previous studies have found that YA men, particularly YA black men, are more likely to misperceive their weight status than their female counterparts.^10^, ^11^, ^12^ In addition, studies in adults of all ages suggest that black men are more likely to underestimate their weight status than white men.^13^, ^14^, ^15^ Although findings are not always consistent,^16^, ^17^ it has been suggested that weight status misperception could lead to a decreased likelihood of attempting weight loss.^18^, ^19^, ^20^ However, the association between weight status perception accuracy and attempted weight loss has not been examined among YA men. Further, to our knowledge, racial differences in weight loss attempts between YA white and black men have not been explored. This lack of information is concerning because weight misperception has increased significantly among YA men over time.^21^ Studies also demonstrate young men are not concerned about weight gain and have to experience significant weight gains to become concerned about their weight.^3^, ^11^, ^14^ Misperceptions of weight status may underlie these associations.

Certain demographic and contextual factors are also known to be significantly related to weight loss attempts, including education, income, number of hours spent working, depressive symptoms, and family medical history. There is limited evidence investigating the influence of these factors among YA men, even though they may be particularly influential during this developmental period.^9^, ^19^ For example, education, income, and working hours may fluctuate during young adulthood and are known to influence health‐related behaviours.^9^, ^19^ Further, depressive symptoms are more prevalent during this period,^22^ with some evidence suggesting that this may negatively impact engagement in weight loss behaviours.^23^ Family medical history also may be a motivating factor for health behaviour change among YAs, particularly for men.^24^, ^25^ Thus, it is important to consider these factors in addition to perceptions of weight status to provide insight into weight loss attempts among YA men with overweight/obesity.

To enhance understanding of factors related to weight loss attempts among YA men, and particularly black men, the present study examined racial differences in the influence of weight status perception accuracy on reported weight loss attempts in the past year among YA men with overweight/obesity. Specifically, it was hypothesized that weight status perception accuracy would be significantly associated with weight loss attempts among YA men and that race would moderate the association between weight status perception accuracy and weight loss attempts. The relative influence of important demographic and contextual factors (ie, income status, education, depressive symptoms, occupational demands, and family medical history) on reported weight loss attempts was also explored.

## 2 MATERIALS AND METHODS

### 2.1 Study design and population

The National Health and Nutrition Examination Survey (NHANES) is an annual survey of the health and nutritional status of the civilian, noninstitutionalized US population.^26^ The survey consists of questionnaires and a physical examination. Data from the 2007 to 2014 survey cycles were used because these were the most recent data available at the time of analysis. The NHANES utilizes a stratified, multistage probability sampling technique to provide a sample representative of the US population. Certain groups, including those identifying as black/African‐American, are oversampled to produce reliable statistical estimates.

The sample for this study was limited to black and white YA men with overweight/obesity between the ages of 20 and 39 years. Young adulthood was defined as 20 to 39 years given the NHANES analytic guidelines, which recommend utilizing this age range because of the sample design. Previous studies focused on YAs using NHANES data used this definition.^4^, ^27^ Analyses were limited to black and white YA men given the focus of this work on understanding racial differences between these understudied groups on weight status perception accuracy and weight loss attempts. Further, only participants with overweight/obesity were included in analyses. This enabled focusing on individuals who would most benefit from weight loss but may not attempt it because of weight misperception. Institutional Review Board review or approval was not required by Temple University because the NHANES dataset is de‐identified and publicly available.

### 2.2 Measures

#### 2.2.1 Demographic characteristics

Key demographic variables assessed in NHANES include the participant's sex, age, race, education level, and income status. Because of a small proportion of the study population reporting less than a 9th‐grade education, this variable was recoded into the following categories: Less than a high school graduate, high school graduate, some college, or college graduate and above. Family income to poverty ratio was used to measure income status. Specifically, the family (or individual) income is divided by the Department of Health and Human Services poverty guidelines for the specific survey year. A value below 1 indicates a family or individual is living below the federal poverty line.^28^

#### 2.2.2 Contextual factors

Relevant contextual factors measured in NHANES included number of hours worked, depressive symptoms, and family medical history. Number of hours worked was assessed by a single item asking “How many hours did you work last week at all jobs or businesses?” Depressive symptoms were assessed with the Patient Health Questionnaire, which is a valid and reliable nine‐item questionnaire that screens for depressive symptoms over the past 2 weeks.^29^ Family history of cardiovascular disease (CVD) and diabetes were assessed with two dichotomous items, which asked if a biological relative was ever diagnosed with a heart attack/angina (CVD) or diabetes, respectively.

#### 2.2.3 Weight, height, and BMI

Height and weight were objectively measured by trained researchers using a calibrated stadiometer and scale, respectively.^27^ BMI (kilograms per square meter) was then calculated. Overweight and obesity were defined according to clinical guidelines.^30^, ^31^ Participants with a measured BMI 25.0 to 29.9 kg/m^2^ or greater than or equal to 30.0 kg/m^2^ were classified as having overweight or obesity, respectively. The dichotomous weight misperception variable was coded on the basis of calculated BMI and participant perception of weight status as underweight, about the right weight, or overweight. If a participant reported perceiving themselves as “underweight” or “about the right weight” when their measured BMI indicated they have overweight/obesity, the dichotomous weight misperception variable was coded “yes.”

#### 2.2.4 Weight loss attempts

Two items on the Weight History Questionnaire were used to create a measure of weight loss attempts in the past year. Specifically, if participants self‐reported a weight from 1 year ago that is 10 pounds or more than their self‐reported current weight, they are asked item (1), “Was the change between your current weight and your weight a year ago intentional?” If a participant does not meet this criterion or responds “No” to this item, they are asked item (2), “During the past 12 months, have you tried to lose weight?” Participants who responded “Yes” to item (1) were not asked item (2), so duplication of participant responses is not a concern. Responses to item (1) are combined with responses to item (2) to create the weight loss attempts primary outcome.

#### 2.2.5 Healthy weight loss behaviours

Given focus of this work on the association between weight status perception accuracy and clinically recommended weight loss strategies,^30^ additional questions from the Weight History Questionnaire were used to focus analyses on YA men who reported engaging in healthy weight loss behaviours relative to those who did not report a weight loss attempt in the past year. Specifically, participants who reported a weight loss attempt were asked additional questions on the Weight History Questionnaire regarding specific weight loss behaviours. Using a coding scheme developed by Chaitoff et al,^32^ these were classified as healthy (eg, eating less fat, exercising, and joining a weight loss programme) and unhealthy (eg, skipping meals, taking laxatives or vomiting, and smoking) weight loss behaviours. YA men who reported at least one unhealthy weight loss behaviour were excluded to enable focus on those who reported using at least one healthy weight loss behaviour relative to those who did not report a weight loss attempt in the past year.

### 2.3 Statistical analysis

Analyses were conducted on weighted NHANES data using Stata/IC 14.2 (College Station, TX: StataCorp LP) svyset and svy procedures to account for the complex sampling design by constructing an appropriate sample design weight according to the analytic guidelines.^27^ Sampling errors were estimated by using the provided primary sampling units and strata and calculated through Taylor series linearization. Associations are identified as statistically significant at the α < .05 level. Univariate descriptive statistics were first run to provide the basic study sample characteristics and assess the amount of missing data. Individual associations between the continuous and categorical predictor variables and the weight loss attempts outcome variable were assessed with *t* tests and Pearson chi‐square tests, respectively. An unadjusted logistic regression first examined the association between accurately perceiving weight status and weight loss attempts in the past year among white and black YA men with overweight/obesity. A second unadjusted logistic regression model then determined the association between weight status perception accuracy and healthy weight loss behaviours among white and black YA men. Factors that were significantly associated with weight loss attempts and weight loss behaviours in preliminary analyses (*P* < .05) were then included in their respective fully adjusted models. Preliminary analyses indicated there was less than 10% missing data for all variables used in the logistic regression models, except for the depressive symptoms variable, which was missing 14% of the data. In accordance with NHANES analytic guidelines,^27^ multiple imputation by chained equations was employed in the fully adjusted models because greater than 10% of the data was missing for depressive symptoms.

## 3 RESULTS

Bivariate associations of study variables for black and white YA men with overweight/obesity are listed in Table 1. In these preliminary analyses, BMI, education level, income status, and depressive symptoms were all associated with reported weight loss attempts (*P* < .05) and were therefore included in the fully adjusted model (Table 2). Number of hours worked (*P* = .91) and family medical history (CVD: *P* = .41; Diabetes: *P* = .74) were not significantly associated with weight loss attempts nor with healthy weight loss behaviours. Although white YA men with overweight/obesity were more likely to perceive themselves as overweight than black YA men (*P* < .01), race was not associated with a reported weight loss attempt nor reported engagement in healthy weight loss behaviours in the past year.

**Table 1:** Bivariate associations of white and black young adult men with overweight/obesity (n = 1417)

Characteristic	White Men (n = 944)	Black Men (n = 473)	*P* Value^a^
Age, y, M (SE)	30.4 (0.22)	30.0 (0.23)	.17
Income status, M (SE)	3.21 (0.08)	2.40 (0.10)	<.01
Education level			<.01
<High school graduate	124 (13.1)	76 (16.1)	
High school graduate	249 (26.4)	146 (30.9)	
Some college	319 (33.8)	172 (36.4)	
College graduate or above	250 (26.5)	79 (16.7)	
BMI, kg/m^2^, M (SE)	30.9 (0.23)	32.5 (0.30)	<.01
Weight status			<.01
Overweight	498 (52.8)	210 (44.4)	
Obese	268 (28.4)	131 (27.7)	
Obese class II	106 (11.2)	80 (16.9)	
Obese class III	72 (7.6)	52 (11.0)	
Close relative had a heart attack			.04
Yes	106 (11.2)	29 (6.1)	
No	813 (86.1)	433 (91.5)	
Close relative had diabetes			<.01
Yes	286 (30.3)	210 (44.4)	
No	636 (67.4)	255 (53.9)	
Number of hours worked last week, M (SE)	36.7 (0.97)	28.7 (1.02)	<.01
Depression score (range 0‐27), M (SE)	2.52 (0.12)	2.56 (0.18)	.86
Weight status perception			<.01
Overweight	584 (61.9)	230 (48.6)	
Underweight	7 (0.7)	11 (2.3)	
About the right weight	351 (37.2)	232 (49.1)	
Weight status perception accuracy			<.01
Inaccurate	358 (37.9)	243 (51.4)	
Accurate	584 (61.9)	230 (48.6)	
Weight loss attempt in the past year			.97
Yes	469 (49.7)	247 (52.2)	
No	475 (50.3)	226 (47.8)	
Healthy weight loss behaviour in the past year			.19
Yes	362 (45.8)	162 (41.5)	
No	475 (54.2)	227 (58.5)	

*Note*. Unweighted counts (column %) are shown unless noted otherwise. Percentages may not sum to 100% and counts may not sum to n because of missing data.

Abbreviations: BMI, body mass index; M, mean; SE, standard error.

a *P* value is from a design‐based Pearson chi‐squared test or *t* test, as appropriate.

**Table 2:** Bivariate associations of predictor variables with weight loss attempts among white and black young adult men with overweight/obesity (n = 1417)

Characteristic	No Weight Loss Attempt (n = 701)	Weight Loss Attempt (n = 716)	*P* Value^a^
Race/ethnicity			.97
Non‐Hispanic white	475 (50.3)	469 (49.7)	
Non‐Hispanic black	226 (47.8)	247 (52.2)	
Age, y, M (SE)	30.5 (0.28)	30.3 (0.22)	.46
Income status, M (SE)	2.94 (0.09)	3.21 (0.08)	.01
Education level			<.01
<High school graduate	131 (65.5)	69 (34.5)	
High school graduate	207 (52.4)	188 (47.6)	
Some college	225 (45.8)	266 (54.2)	
College graduate or above	136 (41.3)	193 (58.7)	
BMI, kg/m^2^, M (SE)	29.9 (0.24)	32. 2 (0.32)	<.01
Close relative had a heart attack			.41
Yes	64 (47.4)	71 (52.6)	
No	624 (50.1)	622 (49.9)	
Close relative had diabetes			.74
Yes	242 (48.8)	254 (51.2)	
No	446 (50.1)	445 (49.9)	
Number of hours worked last week, M (SE)	35.5 (1.25)	35.3 (0.98)	.91
Depressive symptoms (range 0‐27), M (SE)	2.22 (0.16)	2.79 (0.16)	.01
Weight status perception accuracy			<.01
Inaccurate	410 (68.2)	191 (31.8)	
Accurate	290 (35.6)	524 (64.4)	

*Note*. Unweighted counts (row %) are shown unless noted otherwise. Counts may not sum to n because of missing data.

Abbreviations: BMI, body mass index; M, mean; SE, standard error.

a *P* value is from a design‐based Pearson chi‐squared test or *t* test, as appropriate.

**Table 3:** Associations between race, weight status perception accuracy, contextual factors, and weight loss attempts among young adult men (n = 1417)^a^

	Variable	Odds Ratio	95% CI		*P* Value
Unadjusted model	Weight status perception accuracy				
	Inaccurate	Reference	‐‐‐	‐‐‐	‐‐‐
	Accurate	3.73	2.76	5.03	**<.01**
Adjusted model^b^	Weight status perception accuracy				
	Inaccurate	Reference	‐‐‐	‐‐‐	‐‐‐
	Accurate	3.10	2.18	4.41	**<.01**
	Education level				
	<High school graduate	Reference	‐‐‐	‐‐‐	‐‐‐
	High school graduate	1.28	0.84	1.95	.24
	Some college	1.85	1.25	2.74	**<.01**
	College graduate	2.44	1.61	3.72	**<.01**
	Income status	1.10	1.02	1.20	**.02**
	Depressive symptoms	1.06	1.02	1.10	**.01**

*Note*. Boldface indicates statistical significance (*P* < .05).

a Analyses controlled for body mass index (BMI).

b Because of >10% missing data for depressive symptoms, multiple imputation by chained equations was used on all variables.

**Table 4:** Associations between race, weight status perception accuracy, contextual factors, and using healthy weight loss behaviours among young adult men (n = 1417)^a^

	Variable	Odds Ratio	95% CI		*P* Value
Unadjusted model	Weight status perception accuracy				
	Inaccurate	Reference	‐‐‐	‐‐‐	‐‐‐
	Accurate	3.18	2.21	4.59	**<.01**
Adjusted model^b^	Weight status perception accuracy				
	Inaccurate	Reference	‐‐‐	‐‐‐	‐‐‐
	Accurate	3.20	2.16	4.72	**<.01**
	Education level				
	<High school graduate	Reference	‐‐‐	‐‐‐	‐‐‐
	High school graduate	1.08	0.68	1.70	.75
	Some college	1.58	1.00	2.48	**.05**
	College graduate	2.32	1.44	3.74	**<.01**
	Income status	1.10	1.01	1.20	**.04**
	Depressive symptoms	1.02	0.98	1.07	.31

*Note*. Boldface indicates statistical significance (*P* < .05).

a Analyses controlled for body mass index (BMI).

b Because of >10% missing data for depressive symptoms, multiple imputation by chained equations was used on all variables.

Table 3 shows findings from both unadjusted and adjusted logistic regression models for associations between weight status perception accuracy and reported weight loss for YA men with overweight/obesity. In the adjusted model, there was a significant main effect for having an accurate perception of one's weight status (OR = 3.10; 95% CI, 2.18‐4.41; *P* < .01). Participants with some college education or greater had significantly higher odds of reporting a weight loss attempt (OR = 1.85; 95% CI, 1.25‐2.74; *P* < .01) as did individuals reporting higher income (OR = 1.10; 95% CI, 1.02‐1.20; *P* = .02). The adjusted model also revealed a significant association between higher reported depressive symptoms and reporting a weight loss attempt (OR = 1.06; 95% CI, 1.02‐1.10; *P* = .01).

Weight status perception accuracy was also significantly associated with reporting healthy weight loss behaviours in both the unadjusted model (Table 4; OR = 3.18; 95% CI, 2.21‐4.59; *P* < .01) and the model adjusted for contextual factors (OR = 3.20; 95% CI, 2.16‐4.72; *P* < .01). In addition, participants with a college degree were significantly more likely to report using healthy weight loss behaviours (OR = 2.32; 95% CI, 1.44‐3.74; *P* < .01) as were those who reported higher income status (OR = 1.10; 95% CI, 1.01‐1.20; *P* = .04).

## 4 DISCUSSION

In a nationally representative sample of black and white YA men with overweight/obesity, perceiving oneself as having overweight was significantly associated with both reporting a weight loss attempt and reporting engagement in healthy weight loss behaviours in the past year. Specifically, black and white YA men who perceived themselves as having overweight were more than three times as likely to report a weight loss attempt and the use of healthy weight loss behaviours compared with those who did not perceive themselves as having overweight. These findings are consistent with previous work that demonstrated that adults with overweight/obesity who misperceived their weight status were 60% less likely to have tried to lose weight in the past year and that stronger associations between accurate weight perception and weight loss attempts have been observed for adult men relative to women.^18^ Taken together, this work supports the potentially important role of accurate weight status perception for engagement in weight control behaviours, particularly for men.

In contrast to above, prospective studies following adolescents with overweight/obesity into young adulthood produced contradictory findings.^16^, ^17^ Sonneville et al found that adolescents with overweight/obesity who misperceived their weight status had lower BMI gains in young adulthood than their counterparts with an accurate weight perception.^17^ Rancourt et al replicated this finding and further determined that intention to lose weight was not associated with actual BMI change from adolescence to young adulthood.^16^ The authors of both of these studies speculated that these associations could be due to the use of unhealthy weight loss behaviours and experienced weight‐based stigma and teasing (both of which are associated with weight gain)^33^, ^34^ among young people with overweight/obesity who accurately perceived themselves as overweight/obese. As such, developmental differences between adolescents and YAs may partially explain the inconsistency in findings between the current study and prior work. Adolescents may be more sensitive to their weight and shape and may be subject to more weight‐based teasing than adults. These inconsistent findings may also be explained by differences in study designs. The present cross‐sectional study cannot establish the directionality of the relationship between weight status perception and weight loss attempts. Additional longitudinal work in YA populations is needed to determine temporality of associations.

Results from the present study also demonstrate that black young men with overweight/obesity are less likely to perceive themselves as having overweight than white young men with overweight/obesity (49% vs 62%, respectively). This is consistent with studies comparing YA men and women as well as men of all ages.^10^, ^11^, ^12^, ^13^, ^14^, ^15^ Specifically, Gross et al found that only 39% of black young men with overweight/obesity perceived themselves as overweight compared with 68% of black young women with overweight/obesity.^12^ A second study with adults of all ages indicated that black men had a significantly higher likelihood of misperceiving their weight status than white men.^13^ It has been speculated that the higher prevalence of weight status misperception among black men may be explained by sociocultural norms, such as a preference for larger body sizes and reduced pressure to be thin.^12^, ^13^ These sociocultural norms about weight status may be associated with a more positive body image among young black men,^35^ which, in turn, could contribute to young black men being less likely to perceive themselves as having overweight.

Although differences by race were observed for weight status perception accuracy, the present study did not find a significant difference between black and white YA men with overweight/obesity in reported weight loss attempts nor in reported healthy weight loss behaviours in the past year. Thus, race did not moderate the observed association between weight status perception accuracy and reported weight loss. This is in contrast to previous work in adults of all ages that observed racial differences in weight status perception accuracy between black and white men with overweight/obesity, which impacted their respective odds of reporting a weight loss attempt.^15^, ^18^ For example, Dorsey et al found that black men with overweight who misperceived their weight status were less likely to report trying to lose weight than their white counterparts.^15^ However, other than evidence that young men, particularly black men, are less likely to enrol in weight loss programmes,^36^ we are unaware of any studies that have assessed racial differences in independent efforts at weight loss among YA men. The present findings demonstrate there may not be racial differences in weight loss attempts among YA black and white men. These cross‐sectional findings would be enhanced by additional research, including prospective studies to determine if racial differences exist in the understudied group of YA men.

Beyond accuracy of weight perception, certain contextual factors also emerged as significant predictors of reported weight control in the past year. Consistent with the broader adult weight loss literature,^37^, ^38^ higher reported education and income were associated with reported weight loss attempts and healthy weight loss behaviours in the past year. It has been suggested that higher levels of education and those with greater economic means likely have higher levels of health literacy and numerical skills as well as better access to healthy foods and physical activity facilities, all of which help promote healthy weight loss attempts.^39^ Among YAs, high financial strain and credit card debt are significantly associated with low levels of physical activity, excess sedentary behaviour, and unhealthy weight control practices.^40^, ^41^ Reported depressive symptoms, which as a whole, were in the mild range in this sample, were also associated with weight loss attempts but not reported use of healthy weight loss behaviours. It is possible that the mild level of symptoms reported may be reflective of general distress, a factor associated with attempted weight loss in YA men.^42^ Alternatively, given observed associations between depressive symptoms and unhealthy weight loss attempts in YA men,^32^, ^43^ it is also possible that inconsistencies in findings may be due to the fact that the broader measure of weight loss attempts used in the present study also captured participants' use of unhealthy weight loss behaviours.

This study should be considered in light of its strengths and limitations. Strengths include the use of objectively measured heights and weights, which provides a better estimate of weight status than self‐reported heights and weights among YAs.^44^ The use of contemporary data and a large, nationally representative sample are additional strengths, which enhance generalizability to YA men at present. However, the cross‐sectional nature of this study does not allow causal inferences to be made about the association among study variables. Further, participants reported retrospectively on weight loss attempts and behaviours, which could be affected by recall bias. Actual weight loss was not measured in this study, so it is unclear if weight loss attempts resulted in a reduction in weight. It will be important to determine in future studies whether accuracy of weight status perception is associated with subsequent reductions in weight as this could have implications for weight loss interventions. It is also important to note that unhealthy weight loss behaviours were not assessed in this study because of the focus on weight status perception and weight loss attempts among an understudied group—YA men. Associations between weight status perception and unhealthy weight loss behaviours among YA men should be examined in future research. Moreover, using a BMI cut point can also be problematic because participants with a BMI bordering the overweight cut point could be misclassified as having an inaccurate weight status perception because of small differences in weight. However, sensitivity analyses with a higher BMI cut point were conducted and were consistent with the primary findings (data not shown). Weight status perception categories also did not allow for a distinction between overweight and obesity because the question posed in NHANES only allows participants to state if they consider themselves underweight, normal weight, or overweight. Lastly, the fully adjusted model used multiple imputation, which can introduce bias if incorrectly applied.^45^ To ensure the integrity of the findings, the analyses were conducted with and without multiply imputed data, and the results were consistent, suggesting robust associations.

This study extends findings from previous studies conducted with adults of all ages that have demonstrated significant racial differences in weight status perception accuracy and the importance of accurate weight status perception for engagement in weight loss efforts to YA men.^12^, ^13^, ^14^, ^15^, ^18^, ^46^ Findings demonstrate that, among YA men with overweight/obesity, perceiving oneself as overweight is associated with reporting a weight loss attempt and healthy weight loss behaviours in the past year. Reported education, income, and depressive symptoms were also associated with weight loss attempts or specific healthy weight loss behaviours. Although black YA men with overweight/obesity were less likely to accurately perceive themselves as overweight, racial differences between white and black YA men in reported weight loss attempts and healthy weight loss behaviours were not observed. Findings speak to the continued need to identify factors that may account for why YA men, particularly black men, are less likely to report engaging in weight loss efforts.

## CONFLICT OF INTEREST STATEMENT

Dr Sarwer acknowledges consulting relationships with the following companies: BARONova, Ethicon, Merz, and NovoNordisk. Dr. Sarwer is Editor‐in‐Chief of Obesity Science and Practice but was recused from the peer review and editorial decision for this paper. All other authors report no conflicts of interest related to this work.

## FUNDING

This study is from A.P.'s doctoral dissertation and had no direct sources of funding. The authors report no financial disclosures directly related to this work.
